# Immunomodulatory Activity of the Most Commonly Used Antihypertensive Drugs—Angiotensin Converting Enzyme Inhibitors and Angiotensin II Receptor Blockers

**DOI:** 10.3390/ijms23031772

**Published:** 2022-02-04

**Authors:** Paweł Bryniarski, Katarzyna Nazimek, Janusz Marcinkiewicz

**Affiliations:** Department of Immunology, Jagiellonian University Medical College, 18 Czysta Street, 31-121 Krakow, Poland; katarzyna.nazimek@uj.edu.pl (K.N.); janusz.marcinkiewicz@uj.edu.pl (J.M.)

**Keywords:** angiotensin converting enzyme inhibitors, angiotensin II receptor blockers, immunomodulation, immunology, cellular response, humoral response

## Abstract

This review article is focused on antihypertensive drugs, namely angiotensin converting enzyme inhibitors (ACEI) and angiotensin II receptor blockers (ARB), and their immunomodulatory properties reported in hypertensive patients as well as in experimental settings involving studies on animal models and cell lines. The immune regulatory action of ACEI and ARB is mainly connected with the inhibition of proinflammatory cytokine secretion, diminished expression of adhesion molecules, and normalization of CRP concentration in the blood plasma. The topic has significant importance in future medical practice in the therapy of patients with comorbidities with underlying chronic inflammatory responses. Thus, this additional effect of immune regulatory action of ACEI and ARB may also benefit the treatment of patients with metabolic syndrome, allergies, or autoimmune disorders.

## 1. Introduction

Hypertension is a common, chronic disease, which significantly influences the quality of a patient’s life. In a group of US adults (excluding pregnant women) treatment of hypertension was the most popular reason for visiting a doctor’s office and for the chronic use of prescribed medications [1,2]. According to the guidelines of American College of Cardiology/American Heart Association (ACC/AHA), normal blood pressure is below 120 mmHg (systolic)/80 mmHg (diastolic), elevated blood pressure is 120–129/<80 mmHg, while the first stage of hypertension is 130–139/80–89 mmHg and the second stage of hypertension is at least 140/at least 90 mmHg. We can also distinguish isolated systolic hypertension when blood pressure is ≥130/<80 mmHg, and isolated diastolic hypertension when blood pressure is <130/≥80 mmHg [3]. Patients with a blood pressure ≥130/≥80 mmHg are considered to suffer from mixed systolic/diastolic hypertension.

We can also diagnose hypertension by ambulatory blood pressure monitoring (ABPM). Diagnostic criteria according to ACC/AHA guidelines published in 2017 are then as follows: a 24-h mean blood pressure of 125/75 mmHg or above, daytime (awake) mean of 130/80 mmHg or above, and nighttime (asleep) mean of 110/65 mmHg or above [3]. 

According to its pathogenesis, we can divide hypertension into two types: primary (so-called essential hypertension, involving over 90% of cases) and secondary hypertension with known cause. Development of primary hypertension is connected with numerous environmental and genetic factors that have multiple compounding effects on renal and cardiovascular structure and function. The main risk factors related to primary hypertension are age, obesity [4,5], family history [6,7], race [8], reduced nephron number, high-sodium diet, excessive alcohol consumption, and physical inactivity [4,9]. The most common causes of secondary hypertension are primary kidney disease, primary aldosteronism, and sleep apnea syndrome. Less common causes of secondary hypertension include oral contraceptives, pheochromocytoma, Cushing’s syndrome, coarctation of the aorta [10,11], chemiotherapeutic agents and other endocrine disorders (such as hypothyroidism or primary hyperparathyroidism).

Lifestyle modification is essential in all patients with elevated blood pressure or hypertension. These changes should include dietary salt restriction, potassium supplementation, weight loss, exercise, limited alcohol intake, and following the rules of the Dietary Approaches to Stop Hypertension (DASH), which include diet that high in vegetables, fruits, poultry, fish, low-fat dairy products, whole grains, and nuts, and low in sugar-sweetened beverages, red meats, and sweets. However, in most cases pharmacologic therapy is also necessary [12,13,14]. 

Drugs that are appropriate for initial therapy in most patients with hypertension include thiazide-type diuretics, angiotensin-converting enzyme (ACE) inhibitors/angiotensin II receptor blockers (ARBs) and calcium channel blockers. 

Since hypertension is usually associated with a chronic inflammation, examining the effects of drugs on the immune system is a needed research direction in immunology. Resulting knowledge could be then applied to clinical practice, similarly the results of experiments on antidepressant drugs [15,16]. In the precise selection of the drug, the physico-chemical properties of the substance as well as the effects of the substance on the immune system are important.

In this paper, the influence of the most commonly used first-line drugs in the treatment of hypertension, i.e., angiotensin converting enzyme inhibitors and angiotensin II receptor blockers, on the immune system will be discussed.

## 2. Angiotensin Converting Enzyme Inhibitors (ACEI)

### 2.1. Characteristics of the ACEI Drug Group

ACEI inhibit the enzyme that converts angiotensin I to angiotensin II, thereby reducing the concentration of angiotensin II, the vasoconstrictively acting peptide [17]. Due to inhibition of angiotensin conversion, ACEI induce hypotensive, nephroprotective (inhibit proteinuria and progression of renal failure), and antiatherosclerotic effects. ACEI are used especially when, in addition to hypertension, the patient suffers from heart failure or other left ventricular dysfunction (such as hypertrophy or left ventricular malfunction), diabetes or metabolic syndrome, nephropathy, atrial fibrillation or carotid atherosclerosis, or is at a high risk of cardiovascular complications (i.e., stroke or heart attack). Additionally, a previous heart attack or stroke also prompts a recommendation for the use of ACEI. The contraindications to the use of ACEI are pregnancy (these drugs may cause fetal defects) [18], bilateral narrowing of the renal arteries or the unilateral artery stenosis of the only functioning kidney, vascular edema, and hyperkalemia. The most common side effect is cough (caused by an increased concentration of bradykinin) [19,20,21,22], less common are hypotension, hyperkalemia, renal failure, and angioedema [23,24,25,26]. The impact of ACEI on the immune system is shown in Table 1.

### 2.2. Captopril

Captopril (in a dose of 3, 1, or 0.3 micrograms per mL) causes a dose-dependent inhibition of TNF-α synthesis by immune cells. Interestingly, generation of TNF-α by peripheral blood mononuclear cells (PBMC) is increased in patients with chronic heart failure (CHF), especially when accompanied by cachexia, and this effect can be reversed by captopril up to 74% reduction of TNF-α synthesis [27]. At these concentrations, the drug also inhibits the synthesis of IL-1 by 60%, but does not reduce the synthesis of complement C3 by PBMC [28]. However, in another study captopril failed to modulate IL-1beta/IL-2-dependent signaling cascade [29]. The immunomodulatory properties of captopril may contribute to its beneficial effects in heart failure patients [27]. Accumulation of mRNA for IL-1 and TNF is not affected by this drug, suggesting a posttranscriptional effect at a protein level [28,30]. Accordingly, another studies suggest that captopril and lisinopril reduce the release of IL-1beta [31], IL-6, and IL-8 by various cells [32]. A decrease in relative mass and suppression of inflammatory response in the left ventricle, a reduction in plasma levels of IL-1beta and IL-6 and heart expression of their mRNAs, as well as an increase in plasma levels of IL-10 and its mRNA expression in the heart are observed after captopril treatment [33]. In hepatic fibrosis, the use of this medicament has a protective effect as it reduces the levels of the pro-inflammatory cytokine TNF-α and increases the anti-inflammatory cytokine IL-10 in the liver [34]. Captopril enhances IL-10 and IL-2 production by mouse immune cells [35]. In myocarditis caused by clozapine (e.g., indicated in therapy for schizophrenia), captopril reverses the detrimental effect of clozapine on parameters of oxidative stress, such as protection against oxidative DNA damage, production of pro-inflammatory cytokines, and modulation of antioxidant activity [36]. Captopril can also be used in laryngology. This drug used with ARB representative losartan weakened the progression of tympanosclerosis (middle ear sclerosis) by inhibiting TGF-β1 overexpression [37]. Conversely, in psychiatry, chronic treatment with high doses of captopril may cause an increase in plasma IL-1β and IL-6 levels. In addition, this drug may promote depressive behavior by reducing the number of Treg cells and activating microglia [38]. These observations imply that captopril-induced immune effects cause different clinical outcomes depending on patients’ disease history. Accordingly, in cardiovascular disorders, captopril-induced immunomodulation most commonly exerts beneficial effects. In coronary artery disease this medicament decreases IL-6 level, increases TGF-β and IL-22 [39,40], while in acute myocardial infarction it decreases IL-6, TNF-alfa, and C reactive protein (CRP) [41], which in both cases should lead to alleviation of inflammation-related complications. In acute pancreatitis captopril induces a significant decrease in TNF-α concentration in the pancreas, along with decreased MPO activity, NO concentration, and reduction of iNOS gene expression [42]. These observations suggest that administration of captopril before induction of acute pancreatitis suppresses the inflammatory response, which seems to be beneficial in preventing or healing the L-arginine-induced pancreas injury. In addition, after radiation exposure this drug reduces the expression of angiotensin II, inhibits the NF-κB pathway and reduces the overexpression of TGF-β1, protecting the endothelium from radiation-induced injury [43]. This ACEI representative prevents the increase in IL-6, TNF-α, malondialdehyde (MDA), and NO in the hippocampus of rats suffering from experimentally induced memory defects [44]. In the aortic tissue, it clearly reduces the expression of the CD103, CD80, CD86, and MHC-II proteins, while increasing the expression of Foxp3. While used to stimulate splenic dendritic cells, captopril increases IL-10 and TGF-β production, while reducing IL-6 and IL-12 synthesis. This drug inhibits dendritic cell maturation and promotes Treg cell differentiation [45]. In aorta wall tissues captopril reduces the number of infiltrating CCR9+CCL25+ cells, which alleviates the course of atherosclerosis [46]. The synthesis of anti-inflammatory IL-1 receptor antagonist (IL-1RA) is increased by captopril, which induces a systemic beneficial effect on immune reactivity [30]. Additionally, this medicament exerts a dose-dependent immunosuppressive effect on the activity of NK cells in vitro [47].

### 2.3. Cilazapril, Delapril

It was shown that ACEI, such as captopril, delapril, and cilazapril, inhibit TNF-α production in vitro and in vivo when used at high doses [48]. Another report demonstrated that certain ACEI suppress IL-1 and TNF synthesis at a posttranscriptional level and thus could influence cytokine-mediated cell growth [28]. However, these effects were induced only by some ACEI, i.e., enalapril and cilazapril, but not by ramipril, lisinopril, perindopril, or spirapril. This suggests that the effect is not due to the inhibition of angiotensin converting enzyme, but instead results from an additional immune-related activity of some of ACEI, which requires further investigation. 

### 2.4. Lisinopril

Accordingly, lisinopril was shown to decrease levels of IL-6, IL-8 [49], and to inhibit ROI’s production [50]. Similarly to captopril, lisinopril inhibits IL-12 and IFN-gamma production [51]. Altogether, the data suggest that the use of ACEI reduces plasma concentrations of TNF-α and CRP [52], which seems to be responsible for ACEI-induced anti-inflammatory effects in patients with cardiovascular diseases.

### 2.5. Enalapril

While analyzing other immune-related beneficial effects of ACEI, anti-inflammatory activity of enalapril was observed in diabetic nephropathy. Navarro et al. showed that the levels of TNF-α mRNA in renal cortex are doubled in diabetic rats as compared to non-diabetic control animals, and this increase could be prevented by administering enalapril [53]. It is worth adding that daily urinary albumin excretion is correlated with levels of TNF-α in urine and with renal expression of TNF-α, which suggests that enalapril may protect patients with kidney disease against albuminuria [53]. Other studies examining the impact of enalapril on immune system demonstrated that this medicament causes an increase in IL-2 and IL-10 synthesis, which correlates with an increase in the number of CD4+CD103+CD25-spleen-resident T cells [35], and that it significantly increases the number of circulating endothelial progenitor cells after ischemic stress [54]. This drug impacts the humoral immunity as well. It has been shown that enalapril administration significantly increases the production of IgG2c without affecting IgG1 synthesis in mice immunized with ovalbumin [55]. However, enalapril-induced effects seem to depend on mouse sex, and thus likely on the activity of sex hormones, since this drug causes the reduction of production of pro-inflammatory IL-1α, protein-1 monocyte chemoattractant, and macrophage-1a protein in females, and increases the synthesis of anti-inflammatory cytokine IL-10 in males [56]. Additionally, it may affect the intracellular inflammatory signaling cascades. In intestinal epithelial cells and in peritoneal macrophages, enalapril inhibits IκBα phosphorylation and degradation, and reduces NF-κB binding activity, which results in decreased pro-inflammatory cytokine production [57]. In an experimental model of infection with dengue virus, enalapril administration seemed to normalize the levels of IL-1beta and decreased the number of cells expressing viral antigen [58]. In diabetic nephropathy this medicament failed to modulate the B cell-mediated immune response [59], which is contradictory to the previously mentioned impact of enalapril on humoral immunity in healthy mice [55]. On the other hand, treatment with this drug may promote macrophage polarization towards the M1 phenotype [59]. However, in mouse experimental colitis, enalapril significantly reduces TNF-α, IFN-gamma, IL-8, and IL-6 production and thus alleviates the course of intestinal inflammation [60]. This drug attenuates colitis by reducing the infiltration of inflammatory cells in the colon and reducing the expression of pro-inflammatory IL-1β [61]. While this drug failed to affect inflammatory markers in plasma and plaque remodeling in aorta and thus may not prevent thrombosis [62], in inflammatory lung injury enalapril exerted protective effects on the respiratory tract by reducing the concentration of IL-1beta and IL-6 [63]. 

### 2.6. Perindopril

Perindopril inhibits monocyte secretory activity more potently than enalapril and has a stronger anti-inflammatory effect in patients with normal blood pressure and coronary artery disease. Perindopril normalizes the disease-enhanced release of TNF-α, IL-6, IL-1beta, monocyte-1 chemoattractant protein, and CRP [64,65,66]. Similarly, a further report demonstrated that this ACEI representative decreases serum CRP concentration in humans [67], while another study showed that treatment with perindopril causes an increase in IL-10 concentration [66,68], without affecting the levels of IL-4, IL-13, and CRP [68]. This medicament also decreases IL-2 and prevents the unwanted T-cell stimulation [67], as well as inhibits TGF-β1 release in patients with chronic kidney disease [69]. Imidapril and perindopril significantly decrease secretion of TNF-α by human primary monocytes and THP-1 cells [70]. 

### 2.7. Benazepril

Benazepril significantly reduces TNF-α production [71]. In diabetic nephropathy, it significantly reduces NF-κB and TGF-β levels [72], while in left ventricular hypertrophy, benazepril reduces TGF-β, VCAM-1, and NF-κB expression, and ROI’s production. Consequently, this leads to a significant reduction in left ventricular hypertrophy and fibrosis, as well as to an improvement in hemodynamic function [73].

### 2.8. Fosinopril

Similarly, fosinopril, when administered alone or in combination with pravastatin (then producing much stronger effect) has a beneficial effect on left ventricular remodeling after acute myocardial infarction by normalizing elevated matrix metalloproteinase (MMP)-2, MMP-9, and TNF-α levels in the left ventricle [74]. In addition, fosinopril-induced effects were superior to captopril treatment as expressed by significantly better improvement of the overall left ventricular systolic function and stronger reduction of CRP and TNF-α concentrations [75].

### 2.9. Alacepril

Because of having –SH group, alacepril strongly reduces the over-activated production of monocyte chemoattractant protein-1 (MCP-1) and TNF-α and inhibits production of ROIs by human aortic endothelial cells more effectively than lisinopril [50,76].

### 2.10. Zofenopril

Zofenopril also has a sulfhydryl (–SH) group and its administration reduces the level of IL-1beta and decreases expression of CD40 and CD31 that are responsible for recruitment of mononuclear cells and platelets [77]. This drug increases nitric oxide production and its bioactivity [78], but reduces TNF-α levels [79].

### 2.11. Ramipril

In hemodialyzed patients, ramipril increases IL-1beta and decreases IL-10 and IL-6 levels [80]. In young convalescents recovered from aortic coarctation and without elevated blood pressure, ramipril reduces IL-6, sCD40L, and sVCAM-1 levels, but does not affect CRP concentration [81,82]. Ramipril, used in diabetes mellitus type I patients that do not suffer from diabetic nephropathy, does not affect TGF-β and VEGF levels [83].

### 2.12. COVID-19

The impact of ACE inhibitors on the coronavirus infection is ambiguous. On the one hand, ACEIs facilitate the entry of SARS-CoV-2 into cells. On the other hand, they appear to increase the chance of a milder disease course by lowering the concentration of angiotensin II. Increased levels of angiotensin II have been observed in most patients with COVID-19 pulmonary complications. Moreover, the positive hypotensive effect of the ACEI must not be forgotten. Accordingly, at the beginning of the pandemic the European Society of Cardiology advised not to abandon the ACEI administration to patients, by assuming that this action has more pros than cons [84].

**Table 1:** The effect of angiotensin converting enzyme inhibitors (ACEI) on selected parts of the immune system. Abbreviations: TNF-α—tumor necrosis factor alpha; IL—interleukin; NF-κB—nuclear factor kappa-light-chain-enhancer of activated B cells; NO—nitric oxide; iNOS—inducible nitric oxide synthase; MPO—myeloperixidase, TGF—Transforming Growth Factor; CRP—C reactive protein, CD—cluster of differentiation, IFN—interferon, VCAM—vascular cell adhesion protein.

Drug	Immunological Mechanism (Reference)
Captopril	**Reduction in:** - TNF-α synthesis [27,33,41,42,44]; - IL-1 [28]; - the release of IL-1beta [31], IL-6 and IL-8 by various cells [32]; - production of pro-inflammatory cytokines and modulation of antioxidant’s activity [36]; - TGF-β1 overexpression [37,43]; - the number of Treg cells [38]; - C reactive protein (CRP) [41]; - MPO activity, NO concentration and reduction of iNOS gene expression [42] - expression of the CD103, CD80, CD86 and MHC-II proteins [45]; - inhibits dendritic cell maturation [45]; - the number of infiltrating CCR9+CCL25+ cells [46]; - activity of NK cells in vitro [47]. **No significant effect on:** - synthesis of complement C3 [28]; - IL-1beta/IL-2-dependent signaling cascade [29]; - Accumulation of mRNA for IL-1 and TNF [28,30]; **Increase in:** - plasma levels of IL-10 [33,34]; - IL-10 and IL-2 production by mouse immune cells [35]; - TGF-β and IL-22 [39,40]; - promotes Treg cell differentiation [45]; - the synthesis of anti-inflammatory IL-1 receptor antagonist (IL-1RA) [30].
Cilazapril	**Reduction in:** - TNF-α production not only in vitro, but also in vivo, when used at high doses [48].
Delapril	**Reduction in:** - TNF-α production not only in vitro, but also in vivo, when used at high doses [48].
Lisinopril	**Reduction in:** - the release of IL-1beta [31], IL-6 and IL-8 by various cells [32]; - inhibition in ROI’s production [50]; - IL-12 and IFN-gamma production [51]; - plasma concentrations of TNF-α and CRP [52].
Enalapril	**Reduction in:** - TNF-α production [53,60]; - pro-inflammatory IL-1α, protein-1 monocyte chemoattractant and macrophage-1a protein in females [56]; - normalize the levels of IL-1beta [58,61,63]; - IFN-gamma, IL-8 and IL-6 production [60,63]; - colitis by reducing the infiltration of inflammatory cells in the colon [61]; **No significant effect on:** - the B cell-mediated immune response [59]; **Increase in:** - IL-2 and IL-10 synthesis, which correlates with an increase in the number of CD4+CD103+CD25- spleen-resident T cells [35]; - the number of circulating endothelial progenitor cells after ischemic stress [54]; - the production of IgG2c without affecting IgG1 synthesis in mice immunized with ovalbumin [55]; - the synthesis of anti-inflammatory cytokine IL-10 in males mice [56].
Perindopril	**Reduction in:** - monocyte secretory activity [63,64,65,66]; - TNF-α, IL-6, IL-1beta, monocyte-1 chemoattractant protein, and CRP [64,65,66]; - secretion of TNF-α by human primary monocytes and THP-1 cells [70]; - serum CRP concentration in humans [67]; - IL-2 and prevents the unwanted T-cell stimulation [67]; - inhibits TGF-β1 release in patients with chronic kidney disease [69]. **No significant effect on:** - IL-4, IL-13, and CRP [68]. **Increase in:** - IL-10 concentration [66,68].
Benazepril	**Reduction in:** - TNF-α production [71]; - NF-κB and TGF-β levels in diabetic nephropathy [72]; - TGF-β, VCAM-1, and NF-κB expression, and ROI’s production [73]; - left ventricular hypertrophy and fibrosis [73].
Fosinopril	**Reduction in:** - matrix metalloproteinase (MMP)-2, MMP-9 and TNF-α levels in the left ventricle [74]; - CRP concentration [75].
Alacepril	**Reduction in:** - the over-activated production of monocyte chemoattractant protein-1 (MCP-1), TNF-alpha, and production of ROIs by human aortic endothelial cells [50,76].
Zofenopril	**Reduction in:** - IL-1beta and expression of CD40 and CD31 [77]; - TNF-α levels [79]. **Increase in:** - nitric oxide production and its bioactivity [78].
Ramipril	**Reduction in:** - IL-10 and IL-6 levels [80]; - sCD40L and sVCAM-1 levels [81,82]. **No significant effect on:** - CRP concentration [81,82]; - TGF-β and VEGF levels [83]. **Increase in:** - IL-1beta [80].

## 3. Angiotensin II Receptor Blockers (ARBs)

### 3.1. Characteristics of the ARB Drug Group

Hypotensive action of ARBs results from their antagonistic activity against the type 1 angiotensin receptor (AT_1_). The indications and contraindications for use of ARBs are the same as for ACEI, excluding angioedema from the list of contraindications. In addition, ARBs should be used when the patient cannot take ACEI because of a tiring, dry cough. However, so far less is known on the possible immunomodulatory activity of ARBs than of ACEI. The impact of ARBs on the immune system can be seen in Table 2.

### 3.2. Valsartan, Losartan

Valsartan has a strong inhibitory effect on lipopolysaccharide (LPS)-stimulated production of TNF-α and IL-1 by PBMC in vitro. This drug also reduces IL-6 production [30]. Similar effects are induced by candesartan and losartan, which are able to reduce IL-1beta [31], IL-6, and IL-8 concentration [31,32,85]. In acute myocardial infarction, valsartan decreases IL-6, TNF-alfa and CRP [41]. Losartan induces release of TGF-β, which is likely related to its anti-atherosclerotic activity [86]. However, other contradictory data report that losartan administration either decreases the plasma concentration of TGF-β 1 [87], or does not impact the TGF-β 1 serum level and urinary excretion [88]. In coronary artery disease, losartan was shown to decrease IL-6 level, and increase both TGF-β and IL-22 levels [39,40]. In dengue infection, losartan, similarly to enalapril, decreases the number of cells expressing viral antigen and normalizes IL-1beta level [58]. In rheumatoid arthritis, losartan reduces IFN-gamma, IL-6, IL-17F, and IL-22 levels, show strong anti-inflammatory effect, while enalapril and valsartan do not have these properties. Thus, losartan was suggested as a therapeutic agent for patients who suffered from hypertension and rheumatoid arthritis [89]. In hemodialysis patients, valsartan decreases IL-6 concentrations [80]. In addition, valsartan reduces the level of TGF-β1 [90,91]. In rats that underwent myocardial infarction, valsartan decreased Th1 cell numbers and cytokine production, but increased Kir2.1 expression [92]. This drug also potently inhibits the production of IL-1β, IL-6, and TNFα and thereby abolishes the inflammatory activation of macrophages and adipocytes [93]. In mild to moderate essential hypertension, valsartan decreases the disease-increased levels of monocyte/macrophage chemotactic proteins and soluble P-selectin better than indapamide with a similar hypotensive effect [94].

When administered to smokers, losartan normalized the smoking-increased levels of IL-6 [95]. Macrophages co-cultured with losartan poorly produced IL-1beta, but this drug failed to reduce IL-1beta mRNA expression [96]. In different animal models, this drug was found to attenuate inflammation by lowering the levels of IL-6, TNF-α, MCP-1, and IL-1beta [97,98]. Losartan also inhibits M1 macrophage polarization and promotes the shift towards M2 phenotype [99]. Losartan was demonstrated to suppress inflammatory responses by inhibiting Th22 cell chemotaxis in IgA nephropathy [100]. In acute lung injury, losartan inhibits the maturation of dendritic cells accumulated in the respiratory tract, and blocks the Th1 and Th17 polarization of lymphocytes, which leads to a milder disease course [101]. This drug also improves colitis by reducing the infiltration of inflammatory cells [61]. In collagen-induced arthritis, losartan reduces the inflammatory response by inhibiting the MAPK and NF-κB pathways in B and T lymphocytes, which leads to significant alleviation of clinical symptoms. Interestingly, when losartan was administered with a low dose of methotrexate, similar therapeutic effect was achieved, and, importantly, ARB prevented the methotrexate-induced liver and kidney damage [102].

### 3.3. Olmesartan, Telmisartan

In rat model of glomerulonephritis, high doses of olmesartan were demonstrated to reduce both infiltration of CD8+ T cells and activation of M1 macrophages, thus limiting the necrotic lesions. Simultaneously, increase in the number of M2 macrophages and upregulated production of anti-inflammatory cytokines were observed in olmesartan-administered animals [103]. These observations suggest the anti-inflammatory potential of ARBs.

Accordingly, telmisartan therapy seems to induce similar anti-inflammatory effect in various conditions, including hypertension, atherosclerosis, and brain and nervous system disorders. Treatment with telmisartan reduces IL-6, IL-1beta, TNF-α, and MCP-1 levels, inhibits NADPH oxidase activity and ROI production, and decreases the infiltration of CD4+ T cells, likely through acting on peroxisome proliferator-activated receptor gamma (PPARγ) [104,105,106,107,108,109,110]. This drug increases the concentration of anti-inflammatory IL-10 stronger than perindopril, an ACEI representative, without affecting IL-4, IL-13, and CRP levels in hypertensive patients [68]. Furthermore, telmisartan reduced TNF-α, and neutrophil infiltration as well as increased IL-10 in a rat model of ulcerative colitis [111]. After myocardial infarction was induced in rats, this medicament reduced arrhythmias by elevating the level of cardiac connexin 43, likely by inhibiting IL-17 activity [112].

Olmesartan reduces the release of TNF-alfa by macrophages [79,113,114]. Olmesartan, candesartan, and telmisartan administration into mice with collagen-induced arthritis, which is a mouse model of human rheumatoid arthritis, diminished lymphocyte proliferation and IFN-gamma production in vitro assays, and suppressed antigen-specific Th1 and Th2 lymphocytes in vivo. Additionally, olmesartan therapy prevented severe joint destruction in these mice [115]. In rats with hypertension and nephrosclerosis, this medicament significantly reduced renal interstitial fibrosis by lowering the number of infiltrating macrophages [116]. This drug was found more effective in reducing inflammation and protecting myocardial structure and function than an ACEI representative, ramipril [82]. In rats with methotrexate-induced intestinal mucositis, pretreatment with olmesartan suppressed the inflammatory response [117]. However, this beneficial effect was accompanied with an enteropathy of a yet undefined cause. On the other hand, this medicament was found to alleviate intestinal inflammation better than sulfasalazine in a rat model of ulcerative colitis [118]. In a mouse model of Alport syndrome, olmesartan alleviated renal fibrosis by reducing tubular TGFβ expression [119], while in periodontitis it reduced inflammation by lowering IL-1β and TNF-α levels, down-regulating the expression of MMP-2, MMP-9, COX-2, and RANKL, and up-regulating osteoprotegerin [120]. Olmesartan also reversed left ventricular hypertrophy in rats with restorative hypertension by lowering IL-6 levels [121]. 

### 3.4. Candesartan, Irbesartan

Similar antioxidant properties were observed in the case of eprosartan. This drug was found to reduce the neutrophil ability to generate peroxide anions as well as macrophage infiltration [122,123]. Furthermore, candesartan inhibits inflammation by modulating signaling cascades dependent on TNF-α, IL-1beta, IL-2, IL-6, TGF-β, and NF-κB. In addition, it reduces the formation of ROIs by phagocytes and lowers the expression of CD25 and IL-2 release by T cells [113,124,125,126,127,128,129]. However, this medicament seems to not affect the secretion of IL-10 [130], similarly to olmesartan [121]. Candesartan also prevents NF-κB activation by modulating the TLR4 expression, and reduces the release of chemokines by LPS-stimulated human renal tubular epithelial cells [131]. In mice with allergic asthma, administration of candesartan and irbesartan lowered the general number of immune cells in bronchoalveolar lavage fluid and reduced the release of Th2-lymphocyte (IL-4, IL-5 and IL-13) and Th1-lymphocyte (IL-2 and IFN-γ) cytokines [125].

Irbesartan exerts a neuroprotective effect by inhibiting the activation of microglia and macrophages [132]. This medicament reduces the production of IFN-beta and the expression of iNOS, and thus inhibiting NO production [133]. Irbesartan also inhibits the expression of MCP-1 mRNA in THP-1 monocyte cell line stimulated with TNF-α and activates PPARγ [134]. In patients with chronic kidney disease, this drug also modulates the urinary excretion of various cytokines in a dose-dependent manner [135]. Combined therapy with clopidogrel and irbesartan was found to inhibit nephritis by abolishing macrophage infiltration and thus to reduce early kidney damage caused by nephrectomy [136]. There are also some contradictory observations regarding the irbesartan ability to influence the concentration of CRP, TGF-β, TNF-alfa, IL-6, and the expression of NF-κB, ICAM-1, VCAM-1, and MCP-1 [137,138,139,140,141]. 

**Table 2:** The effect of angiotensin II receptor blockers (ARB) on selected parts of the immune system. Abbreviations: TNF-α—tumor necrosis factor alpha; IL—interleukin; NF-κB—nuclear factor kappa-light-chain-enhancer of activated B cells; NO—nitric oxide; iNOS—inducible nitric oxide synthase; TGF—Transforming Growth Factor, CRP—C reactive protein; CD—cluster of differentiation, IFN—interferon.

Drug	Immunological Mechanism (Reference)
Valsartan	**Reduction in:** - TNF—α and IL-1 concentration [33,93]; - IL-6 production [30]; - TGF-β1 concentration [90,91] - Th1 cell numbers [92]; - abolishes the inflammatory activation of macrophages and adipocytes [93]; - levels of monocyte/macrophage chemotactic proteins [94].
Candesartan	**Reduction in:** - IL-1beta [31], IL-6 and IL-8 concentration [31,32,85]; - CRP concentration [41]; - TNF-alfa concentration [41,113,124,125,126,127,128,129]; - lymphocyte proliferation and IFN-gamma production in vitro assays, and suppressed antigen-specific Th1 and Th2 lymphocytes in vivo [115]; - formation of ROIs by phagocytes [113,124,125,126,127,128,129]; - the expression of CD25 and IL-2 release by T cells [113,124,125,126,127,128,129]; - the general number of immune cells in bronchoalveolar lavage fluid [125]; - the release of Th2-lymphocyte (IL-4, IL-5 and IL-13) and Th1-lymphocyte (IL-2 and IFN-γ) cytokines [125]. **No significant effect on:** - the secretion of IL-10 [130].
Losartan	**Reduction in:** - IL-1beta [31], IL-6 and IL-8 concentration [31,32,85]; - the plasma concentration of TGF—β 1 [87]; - IFN-gamma, IL-6, IL-17F and IL-22 [89]; - TNF—α concentration [97,98]; - inhibits M1 macrophage polarization and promotes the shift towards M2 phenotype [99]; - inflammatory responses by inhibiting Th22 cell chemotaxis in IgA nephropathy [100]; - the maturation of dendritic cells accumulated in the respiratory tract, and blocks the Th1 and Th17 polarization of lymphocytes [101]; - the inflammatory response by inhibiting the MAPK and NF-κB pathways in B and T lymphocytes [102]. **No significant effect on:** - TGF—β 1 serum level and urinary excretion [88]. **Increase in:** - both TGF—β and IL-22 levels [39,40].
Olmesartan	**Reduction in:** - infiltration of CD8+ T cells and activated M1 macrophages [103]; - the release of TNF-alfa by macrophages [79,113,114]. - lymphocyte proliferation and IFN-gamma production in vitro assays, and suppressed antigen-specific Th1 and Th2 lymphocytes in vivo [115]; - the number of infiltrating macrophages [116]; - tubular TGFβ expression [119]; - IL-1β and TNF-α levels, down-regulating the expression of MMP-2, MMP-9, COX-2 and RANKL [120]; - IL-6 level [121]. **Increase in:** - in the number of M2 macrophages and upregulated production of anti-inflammatory cytokines [103]; - osteoprotegerin [120].
Eprosartan	**Reduction in:** - the neutrophil ability to generate peroxide anions as well as macrophage infiltration [122,123].
Telmisartan	**Reduction in:** - IL-6, IL-1beta, TNF—α, and MCP-1 levels [97,98,99,100,101,102,103]; - NADPH oxidase activity [104,105,106,107,108,109,110]; - ROI’s production [104,105,106,107,108,109,110]; - the infiltration of CD4+ T cells [104,105,106,107,108,109,110]; -IL-17 activity [112]; - lymphocyte proliferation and IFN-gamma production in vitro assays, and suppressed antigen-specific Th1 and Th2 lymphocytes in vivo [115]. **No significant effect on:** - IL-4, IL-13, and CRP levels in hypertensive patients [111]; **Increase in:** - concentration of anti-inflammatory IL-10 [111].
Irbesartan	**Reduction in:** - the general number of immune cells in bronchoalveolar lavage fluid [125]; - the release of Th2-lymphocyte (IL-4, IL-5 and IL-13) and Th1-lymphocyte (IL-2 and IFN-γ) cytokines [125]; - inhibiting the activation of microglia and macrophages [132]; - the production of IFN-beta and the expression of iNOS, and thus inhibits NO production [133]; - expression of MCP-1 mRNA in THP-1 monocyte cell line stimulated with TNF-α and activates PPARγ [134]; - macrophage infiltration [136].

## 4. The Most Recent Studies

Recent studies of antihypertensive drugs (diuretics (furosemide, hydrochlorothiazide), ACEI, and combination drugs (ACEI + diuretic) that were conducted in CBA mice showed that diuretics administered alone or with captopril increase the generation of Reactive Oxygen Intermediates, but reduce the formation of NO by macrophages and also increase the expression of surface markers important for the phagocytosis process (CD11b, CD16/32, CDC14) and antigen presentation (CD80, CD86, CD40, I-Ak). Furosemide and hydrochlorothiazide treatment increase generation of activated B cell SRBCs (early humoral response). Captopril does not affect the early response, but when added to furosemide it enhances the early humoral response, and when added to hydrochlorothiazide it reduces it. Captopril (such as furosemide and hydrochlorothiazide) enhances the maturation of antibodies through switching classes. Furosemide added to captopril enhances its effect, while hydrochlorothiazide added to captopril does not [142].

In the cellular response in the antigen presentation phase in the transfer of hapten-labeled macrophages, treatment with all single drugs reduces the presentation activity. Adding captopril to diuretics does not change the activity of the presentation. On the other hand, in the phase of induction of the cellular response in active sensitization with hapten, all drugs significantly reduce the contact hypersensitivity reaction in relation to the control. In the induction of a cellular response in the transfer of effector cells in the delayed-type hypersensitivity captopril and furosemide strongly inhibit the contact hypersensitivity reaction and hydrochlorothiazide has no influence on the reaction. Diuretics with or without captopril modulate humoral and allergic cellular responses by affecting macrophage function [143].

Most of diuretics change the immune response, modulating it towards the anti-inflammatory response [144].

**Figure 1:**
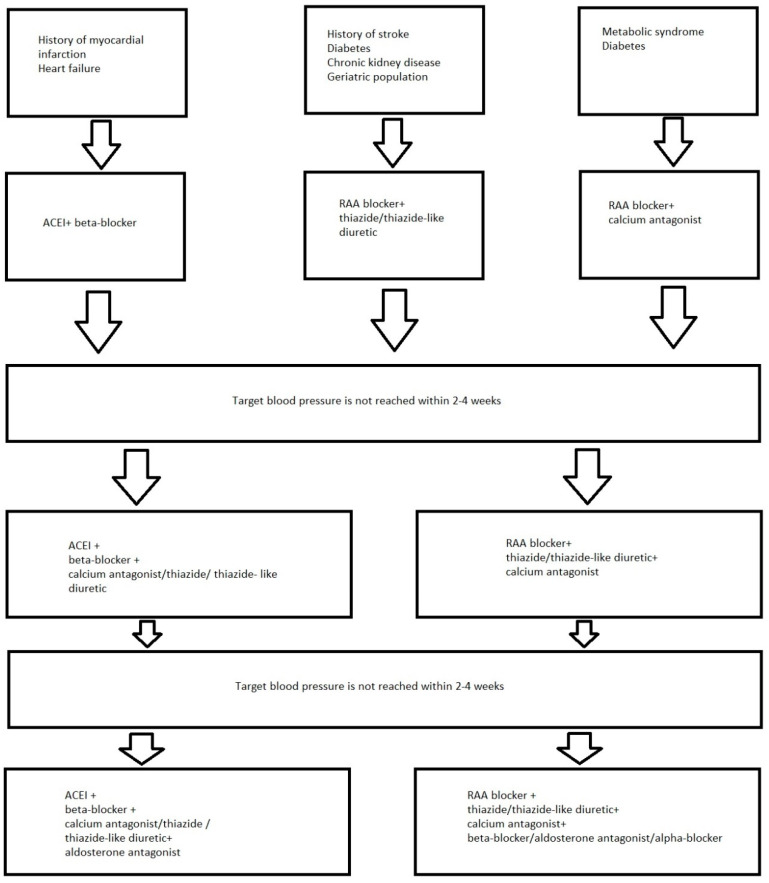
Recommendations for the use of drugs from the appropriate drug groups depending on the diseases accompanying hypertension. Abbreviation: RAA blocker—renin-angiotensin-aldosterone system blocker.

Recommendations for the use of drugs from the appropriate drug groups depending on the diseases accompanying arterial hypertension are presented in Figure 1 [145].

## 5. Conclusions

ACEI and ARBs, the most commonly used antihypertensive drugs, significantly impact the functions of immune cells, and modulate the mechanisms of immune response not only in hypertensive patients, but also in people with immune-related and inflammatory diseases, and even in healthy subjects (Table 1). Therefore, the immunomodulatory properties of ACEI and ARBs are often used in other inflammatory diseases. The use of ACEI and ARB in combination with antihypertensive drugs from other classes multiplies the beneficial systemic therapeutic effect in relieving chronic inflammation. However, it is worth remembering to achieve a balance between the anti-inflammatory component and protection against cancer and microbes in the treatment of inflammatory diseases.

## Data Availability

All data are included within the manuscript.
